# A single cell transcriptomic resource of murine implantation sites at embryonic days 6.5 and 10.5

**DOI:** 10.1093/biolre/ioaf155

**Published:** 2025-07-11

**Authors:** Juan F Garcia Rivas, Wendy Duan, Bethan Wilson, Meghan Riddell

**Affiliations:** Department of Obstetrics and Gynecology, University of Alberta, Alberta, Edmonton, Canada; Department of Physiology, University of Alberta, Alberta, Edmonton, Canada; Department of Physiology, University of Alberta, Alberta, Edmonton, Canada; Department of Obstetrics and Gynecology, University of Alberta, Alberta, Edmonton, Canada; Department of Physiology, University of Alberta, Alberta, Edmonton, Canada

Dear Editor,

Maternal adaptations are a hallmark of pregnancy and are required for the proper nurturing of a developing embryo. Since a myriad of changes support a successful pregnancy, interactions that occur at the maternal-fetal interface are paramount. In mice, orchestration of these events occurs in a brief period, as mouse gestation is ~21 days. Because of this, the temporal differences between early and mid-gestation are of great importance, as supporting a growing embryo is a dynamic process. In mice, decidualization of the endometrium occurs after implantation, initiating a rapid series of adaptations. Maternal decidual adaptations that are seen early in gestation include the expansion of immune cells, decidualization of stromal cells due to an increase in progesterone, and peak spiral artery remodeling mid-gestation [1, 2]. Parallel to maternal adaptations, early embryogenesis includes critical processes like gastrulation and eventually organogenesis [3]. The sum of these interconnected processes is what support a viable pregnancy. Murine embryogenesis and pregnancy are intensely studied, but historically this knowledge has been acquired by analyzing the role of a single molecule at a time. Single cell RNA-sequencing (scRNA-seq) allows for the comparison of multiple cell populations in various cell states simultaneously. This can be used to better understand time-dependent changes in cellular composition and gene expression. Here, we present scRNA-seq analyses on murine implantation sites at gestational day (GD) 6.5 and 10.5 and examine the temporal changes that occur from early to mid-gestation. These analyses were conducted on C57BL/6J mice to serve as a resource to the community. We also made an interactive webpage with these datasets, providing a way for anyone to further explore genes and cell types of interest without the need for coding skills. The interactive dataset can be found at http://riddell-lab.shinyapps.io/MouseSingleCell

Our integrated dataset contains 7105 cells, from four implantations site collected from three dams. Analyses and preprocessing were performed using the standard 10X genomics pipeline followed by Seurat. The GD 6.5 dataset (Supplemental Figure S1) consists of 1466 cells. As seen in Figure 1A the highest proportion of cells are immune cells, making up almost 40% of our dataset, which were identified by their expression of *Ptprc*. Immune cells were further subclassified into uterine natural killer (uNK) cells, T cells and macrophages, based on expression of *Ncr1*, *Cd3d,* and *Cd86,* respectively. The biggest cluster in this dataset was the stromal cluster, marked by the expression of *Dcn*. Embryonic ectoderm and erythroblasts were also highly abundant. These cell populations match what has previously been reported, as proliferation of uNK cells starts at GD 5.5–6.5 in the decidua to prepare for spiral artery remodeling [4]. Gastrulation and formation of the primitive streak contribute to the high proportion of ectodermal cells. As expected, a high proportion of immune cells and stromal cells help to support the developing conceptus.

**Figure 1:**
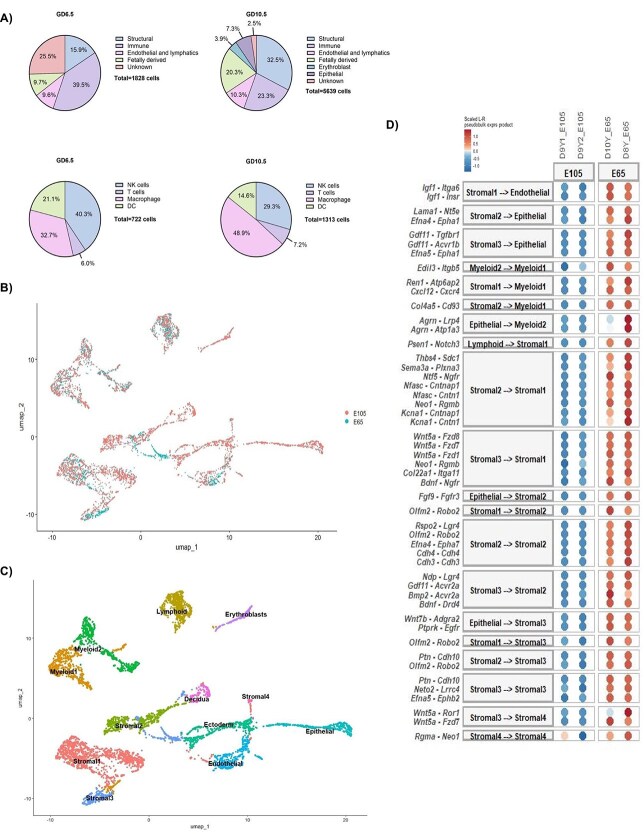
Single cell transcriptomic analysis reveals key differences during early and mid-gestation. (A) Pie charts showing the differences in cell distribution between GD 6.5 and 10.5. (B) Dimensional reduction plot showcasing the cells present in the integrated dataset by gestational age. (C) Uniform Manifold Approximation and Projection plot for the integrated data set, which is made up of 7105 cells and 12 different clusters. (D) Dot plot showcasing the top 50 results from the MultiNichenet analysis; upregulated interactions (red); downregulated interactions (blue).

The GD 10.5 dataset (Supplemental Figure S2) contains 5639 cells. Compared to GD 6.5 there is a shift in the decidual composition, resulting in a higher proportion of stromal cells, from 16% to 32%, compared to immune cells, which made up slightly less than one fourth of the cells at GD 10.5, at 23.3%. There was also a higher proportion of endothelial cells that are not as common earlier in gestation (Figure 1A and B).

When integrating the datasets, the patterns seen at GD 6.5 and GD 10.5 are also seen in our integrated dataset, with stromal and immune cells clusters making up most of the cells (Figure 1C). However, it is important to note that integrating the datasets highlights the presence of some cell clusters that only come from either the GD 6.5 or GD 10.5 samples. For example, many cells seen in the stromal 3 cluster in the integrated dataset come from GD 6.5, highlighting the role that some cells might have early during gestation but not at later timepoints. On the contrary, the presence of actively decidualizing cells, which are marked by genes *Mug1* and *Lama5*, are only seen at GD 10.5, and further supports the concept that decidualization is an ongoing process during mid-gestation, highlighting the temporal changes seen as gestation progresses. Finally, the decidualized cell cluster, which was identified by the expression of a variety of prolactin genes, including *Prl8a2*, is also only seen at GD 10.5. This is expected as decidualization peaks around mid-gestation during murine development [5]. Integrating the data revealed the dynamic nature of the maternal fetal interface across different gestational timepoints and the temporal changes that both the maternal and fetal tissue undergo to properly support a viable pregnancy.

We ran a MultiNichenet [6] analysis with the integrated dataset to interpolate the changes in cell–cell communication that are present between the gestational time points (Figure 1D). Out of the 71 genes and 50 interactions that appeared, almost a third of the genes were previously identified to cause embryonically lethality, cause major developmental issues, or cause female infertility in transgenic knockout mouse models. Some examples of this are *Gdf1, Acvr1b*, and *Cxcl12* interacting with *Cxcr4* [7, 8]. For the remaining interactions, knockout mouse models for many of the genes have no reported developmental or reproductive phenotypes, or the genes have not been adequately studied in murine pregnancy and development. For instance, the interaction between *Thb4* and *Sdc1*, an interaction that has been previously identified in tendon formation and healing, but its role during development is poorly understood [9]. It is important to note that our MultiNichenet analysis was biased toward identification of interactions that were upregulated at GD 6.5 compared to 10.5. This may be because of the ongoing processes of decidualization, gastrulation and organogenesis that are seen during early to mid-gestation.

Here, we provide a resource for the community of an interactive dataset of maternal fetal interface development at GD 6.5 and 10.5 and an integrated dataset of both timepoints. To our understanding, this is the first single set dataset that contains both gestational ages, providing a useful resource to better understand pregnancy adaptations in the maternal fetal interface at early and mid-gestation.

## Methods

### Animal care and breeding

All experiments were approved by the University of Alberta Research Ethics Committee and were performed in accordance with the Canadian Council on Animal Care guidelines. C57BL/6J mice were obtained from The Jackson Laboratory (Bar Harbor, ME, USA). The animals were housed in the University of Alberta conventional animal facility, under standard environmental conditions. To generate timed pregnancies, one male mouse and two female mice were set in a cage after 16:00 h and left overnight. The following morning at 9:00 h the presence of a vaginal plug was examined to assess for copulation. Each cage was left for a maximum of two nights. After a vaginal plug was seen, noon of that day was considered GD 0.5.

### Dam dissection and embryo collection

Dams at either GD 6.5 or 10.5 were euthanized via anesthesia using isoflurane inhalation immediately followed by cardiac puncturing. An abdominal incision was made, and the entire uterus was removed. For GD 10.5, an incision at each implantation site was made to remove the uterus, uterine lining, placenta, and embryo.

### Cell dissociation and suspension

The freshly dissected gestational sacs were dissociated using 1 mg/mL of Collagenase type II [Worthington Biochemical (Lakewood NJ); LS004176] dissolved in DMEM [Gibco (Grand Island, NY); 11,965–092]. Four serial digestions of 30 min at 37°C a rotating wheel were performed; the first one utilizing twice the volume of dissociating media compared to the tissue, with the remaining three being a 1:1 ratio of tissue and collagenase solution. Cells were suspended in Cellbanker 1 [Nippon Zenyaku Kogyo Ltd. (Fukushima, Japan); Lot 231,012] and cryopreserved until single cell sequencing sample processing. Single cell partitioning and library generation for sequencing were prepared using the Chromium next GEM Single Cell 3′ Gene Expression v3.1 kit (10× Genomics). Library preparation was performed by the Advanced Cell Exploration core at the University of Alberta, and sequencing was conducted by Novogene via Illumina Next-Generation sequencing (Sacramento CA).

### Single cell analysis

Feature-barcode matrices, read alignment and other analyses were performed utilizing the CellRanger software (10× Genomics). A total of 7105 cells were in our integrated dataset. Data clustering and visualization was performed in two different software packages. Clustering analysis was performed using Seurat V4 in R. Filtering in Seurat was done by features, with cells being excluded if they had ˂200 or ˃2500 features, as well as mitochondrial counts (percent. mt <35). Identity of the different clusters was determined utilizing a two-step method: (i) gene ontology analysis [10] to determine the top biological processes associated with each cluster and (ii) expression analysis of known features for certain cell types.

## Grant support

None declared.

## Supplementary Material

Supplemental_figures_ioaf155

## Data Availability

The data for this project are available at NCBI GEO under the name “Single Cell Sequencing of Mouse Embryo at Early and Mid Gestation” and the accession number “GSE299135”.
