# Supplementary material for: A single cell transcriptomic resource of murine implantation sites at embryonic days 6.5 and 10.5
Source: Biol Reprod. 2025 Jul 11;113(3):487–90. doi: 10.1093/biolre/ioaf155 (PMC12448632; doi:10.1093/biolre/ioaf155)
Supplement: Supplemental_figures_ioaf155 [file supplemental_figures_ioaf155.pptx]

## Slide 1
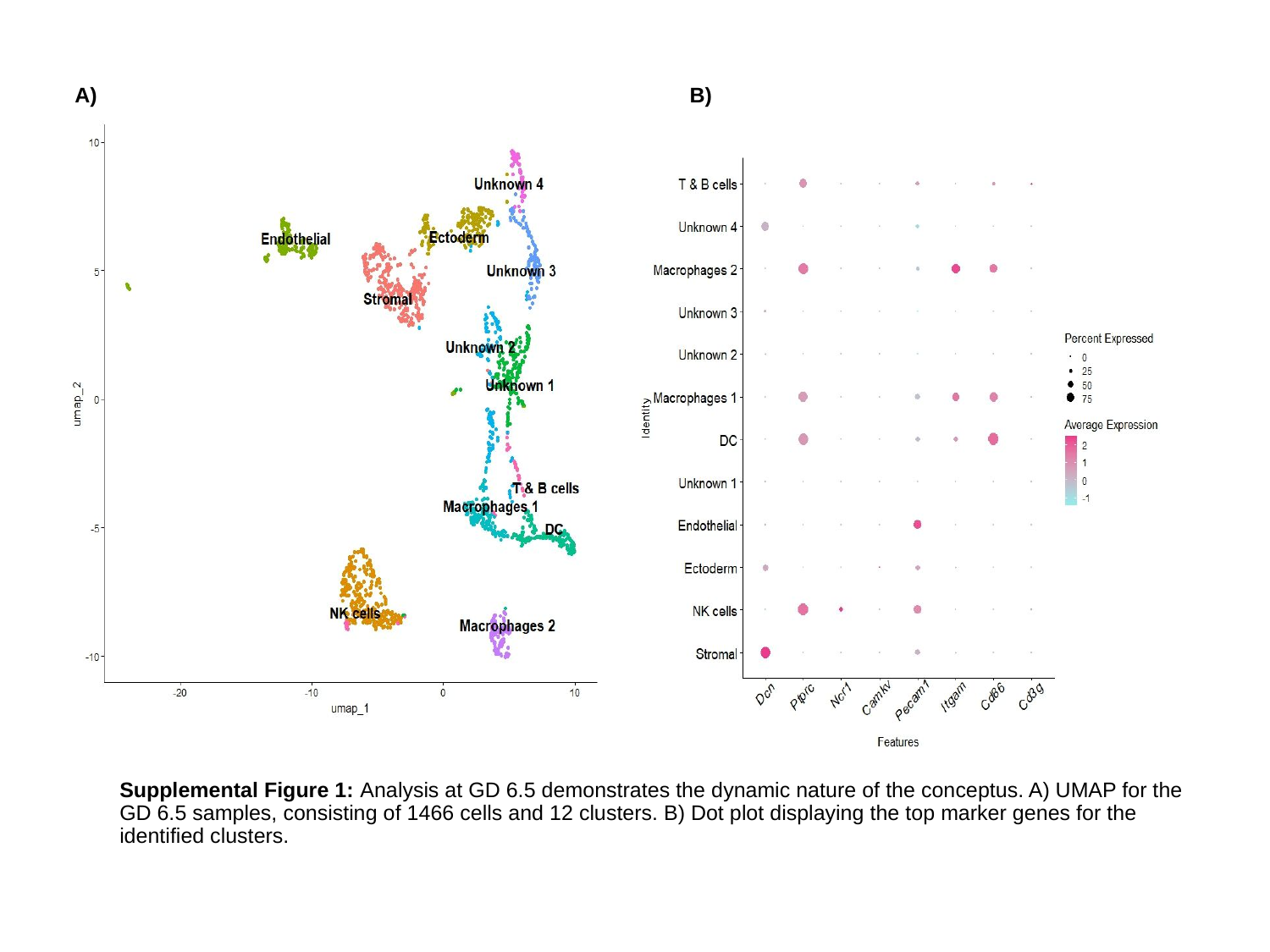

A)
B)
Supplemental Figure 1: Analysis at GD 6.5 demonstrates the dynamic nature of the conceptus. A) UMAP for the GD 6.5 samples, consisting of 1466 cells and 12 clusters. B) Dot plot displaying the top marker genes for the identified clusters.

## Slide 2
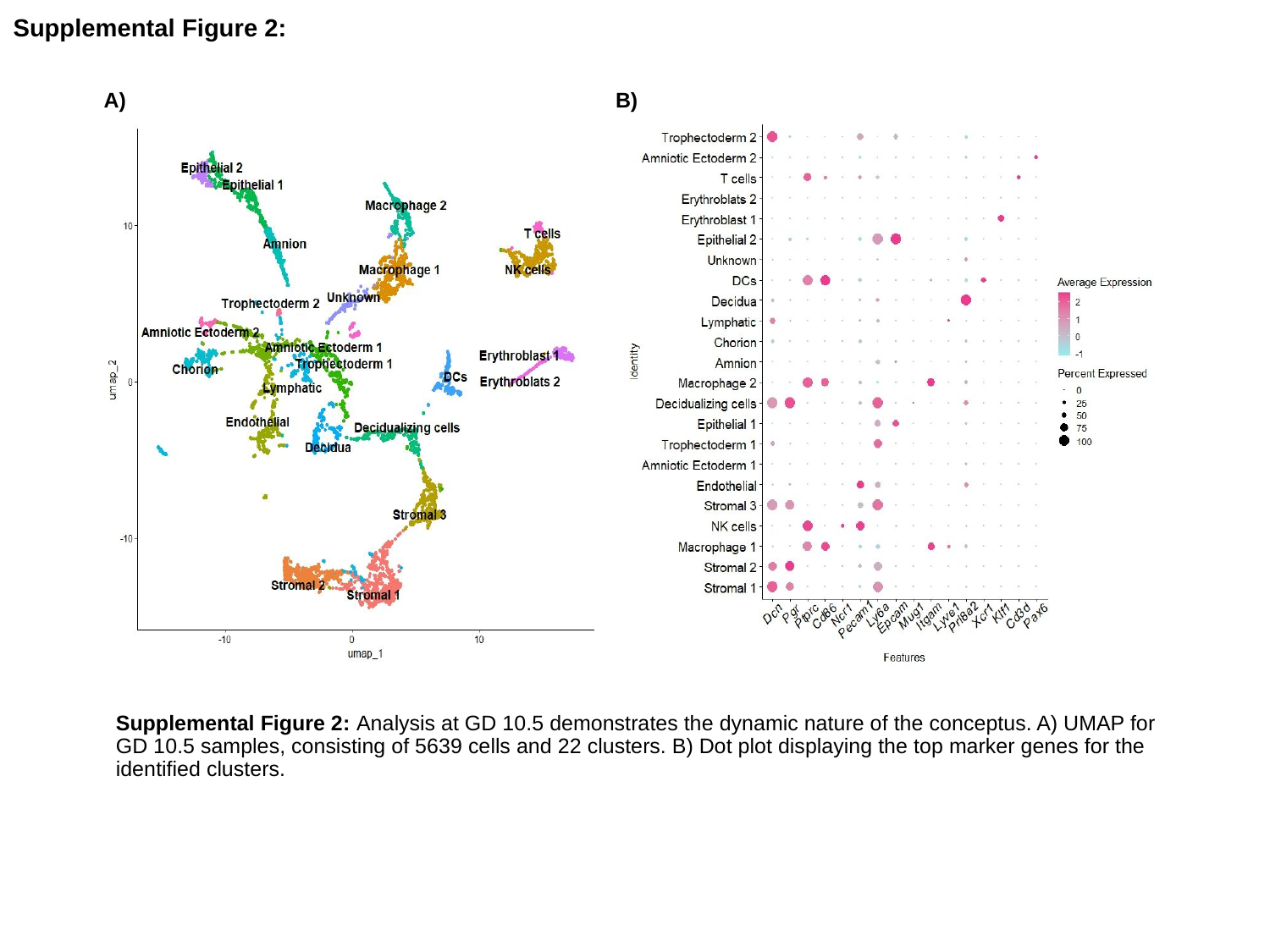

Supplemental Figure 2:
A)
B)
Supplemental Figure 2: Analysis at GD 10.5 demonstrates the dynamic nature of the conceptus. A) UMAP for GD 10.5 samples, consisting of 5639 cells and 22 clusters. B) Dot plot displaying the top marker genes for the identified clusters.
